# A randomized clinical trial of dentin hypersensitivity reduction over one month after a single topical application of comparable materials

**DOI:** 10.1038/s41598-021-86258-3

**Published:** 2021-03-24

**Authors:** Samar Hatem Abuzinadah, Abdulrahman Jafar Alhaddad

**Affiliations:** 1 Department of Restorative Dentistry, Faculty of Dentistry, King Abdul-Aziz University, Alehtifalat street, Jeddah, 21589 Saudi Arabia; 2 Department of Oral and Maxillofacial Prosthodontics, Faculty of Dentistry, King Abdul-Aziz University, Alehtifalat street, Jeddah, 21589 Saudi Arabia

**Keywords:** Health occupations, Materials science

## Abstract

Dentinal hypersensitivity (DH) is a condition that causes patient discomfort. To evaluate the clinical efficacy of Gluma, fluoride varnish and Tetric N-Bond self-etch system in relieving DH immediately and over 30 days following a single topical application. The present randomized clinical trial was conducted on 55 patients with an age range 20–49 years. 70 teeth in total were incorporated and randomly assigned to the three groups. Parameters examined were: Tactile, air blast, and cold stimuli. VAS was used to assess tactile stimulus whereas the Schiff Cold Scale for air blast and cold stimuli. DH was evaluated immediately, at two weeks and 1 month follow up. Gluma showed a statistically significant reduction in DH scores over other materials. It was concluded that Gluma have statistically significant results over other materials in relieving DH immediately and over 30 days following a single topical application.

**Trial Registration:** Clinical Trials.gov Identifier: NCT04351412, King Abdulaziz University Protocol Record 129-09-19. Registered 17 April 2020 – Retrospectively registered. http://ClinicalTrials.gov/NCT04351412.

## Introduction

**Figure 1:**
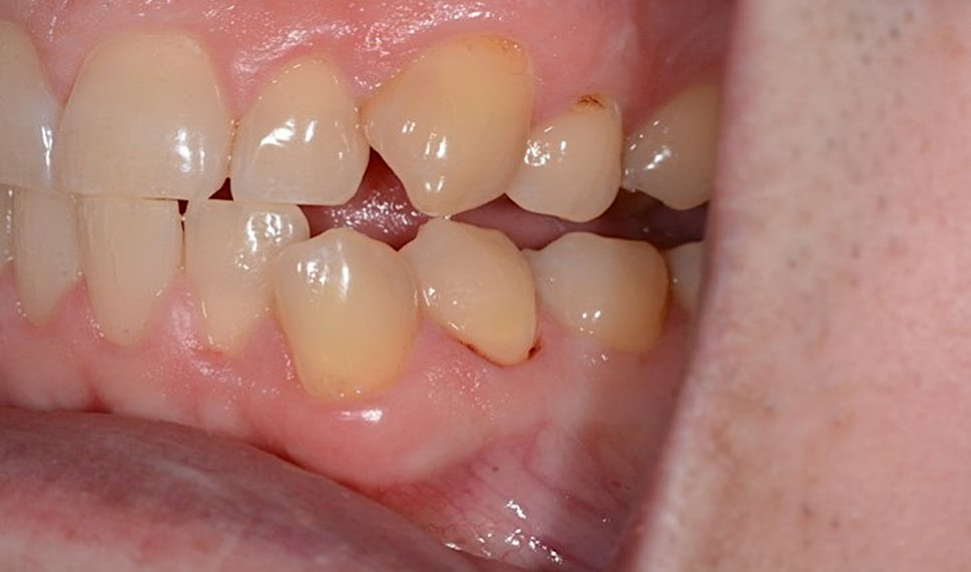
Patient with exposed cervical dentin surfaces and signs of erosion.

Dentinal hypersensitivity (DH) is a condition commonly encountered in dental practice, particularly in individuals who suffer from abrasion, recession of the gingiva, and tooth erosion. DH causes patient discomfort and hampers everyday activities. It is distinguished by sharp, stabbing pain caused by exposed cervical dentin^1^. Dentinal hypersensitivity pain is aggravated in response to patent dentinal tubules when responding to provocation, such as temperature changes, touch, osmotic, chemical, or dispersal that cannot be explained by other conditions (Fig. 1)^2^.

It commonly affects adults, mainly in their 30 s, although it can have an impact on people aged 29 to 49 years. It occurs in up to 47% of the general population and in markedly more among periodontal patients^3^. The buccogingival areas of the canine- and premolar-sextant anterior locations that are prone to gingival recession are often affected by DH^4,5^. The well-known, widely disseminated explanation for tooth DH is Brännström and Åström's hydrodynamic theory^6^. Based on their theory, when the patent dentinal tubules experience external stimulation, it causes movement of the intra-dentinal tubular fluid, stimulating the intratubular nerve endings, and generating discomfort. The number, size, and diameter of the patent dentinal tubules determine the level of sensitivity experienced by individuals^7^.

Until recently, two approaches were used to cure cervical dentinal sensitivity (CDS). The first is blocking the dentinal tubules, and the second is intervening in the response of the mechanoreceptors^8^. Various materials have been manufactured to relieve the pain arising from the cervical dentin sensitivity^9^. The best treatment for DH is typically a desensitizing toothpaste. Many patients experience interim relief when using this on a regular basis over many weeks. Self-applied desensitizing materials have the advantage of being instantly available, unlike treatment options applied by a professional. Most commonly used agents that can be applied by the patients themselves contain a potassium compound, but it has the drawback that it takes a long time to experience symptomatic relief (typically 14 days to 1 month, and up to 3 months)^10^.

Gluma consists of glutardialdehyde-hydroxyethyl-methacrylate and prevents hypersensitivity by reducing dentinal permeability and clotting the proteins and amino acids in the peripheral dentinal tubules^11^. This inhibits the flow of fluid through the tubules, which is the cause of the sensitivity. Patients can also experience sensitivity after invasive restorative work. Gluma desensitizer (Heraeus Kulzer, Armonk, NY, USA) can be used after tooth preparation for receiving indirect restorations and below every restoration to relieve sensitivity and ensure comfort. Gluma restores collapsed collagenous fibers, thereby improving the bond strength of many adhesives.

Some reports have also proposed that self-etch dental bonding agents can decrease DH by creating an acid-resistant hybrid layer. The hybrid layer can decrease DH by reducing dentin permeability^12–14^. The consequence of this acid–base resistance zone is a prolonged enduring clinical effectiveness. The one-step self-etch system, or all-in-one adhesives, which are seventh generation adhesives, are characterized by a self-etch, acidic primer with adhesive resin in a single bottle, which is applied in a single step. This special feature makes it possible for the adhesive resin to infiltrate to the depth of demineralization, which may be how it eliminates postoperative sensitivity. To support this theory, Askari and Yazdani in 2019 compared propolis extract desensitizing agents to Single Bond Universal dentin bonding agent. They concluded that the dentin bonding agent was effective in relieving DH in the long-term and that using Single Bond Universal results in rapid relief of DH^1^.

The present trial was conducted to compare the clinical efficacy of Gluma (Heraeus Kulzer), fluoride varnish, and Tetric N-Bond one-step self-etch system (Ivoclar Vivadent, Schaan, Liechtenstein) in relieving DH immediately and over a 30-day period following a single topical application. The hypothesis was that the effects of Gluma would be statistically significantly different from those of fluoride varnish and Tetric N-Bond one-step self-etch system.

## Materials and methods

This study was designed as a randomized, double blind, controlled clinical trial conducted at King Abdulaziz University dental hospital, Jeddah, Saudi Arabia from September 2019 till February 2020. The study was approved by the institutional review board of research ethical committee. Digitally signed consent was obtained from all participants. No limitations were addressed during the study.

**Figure 2:**
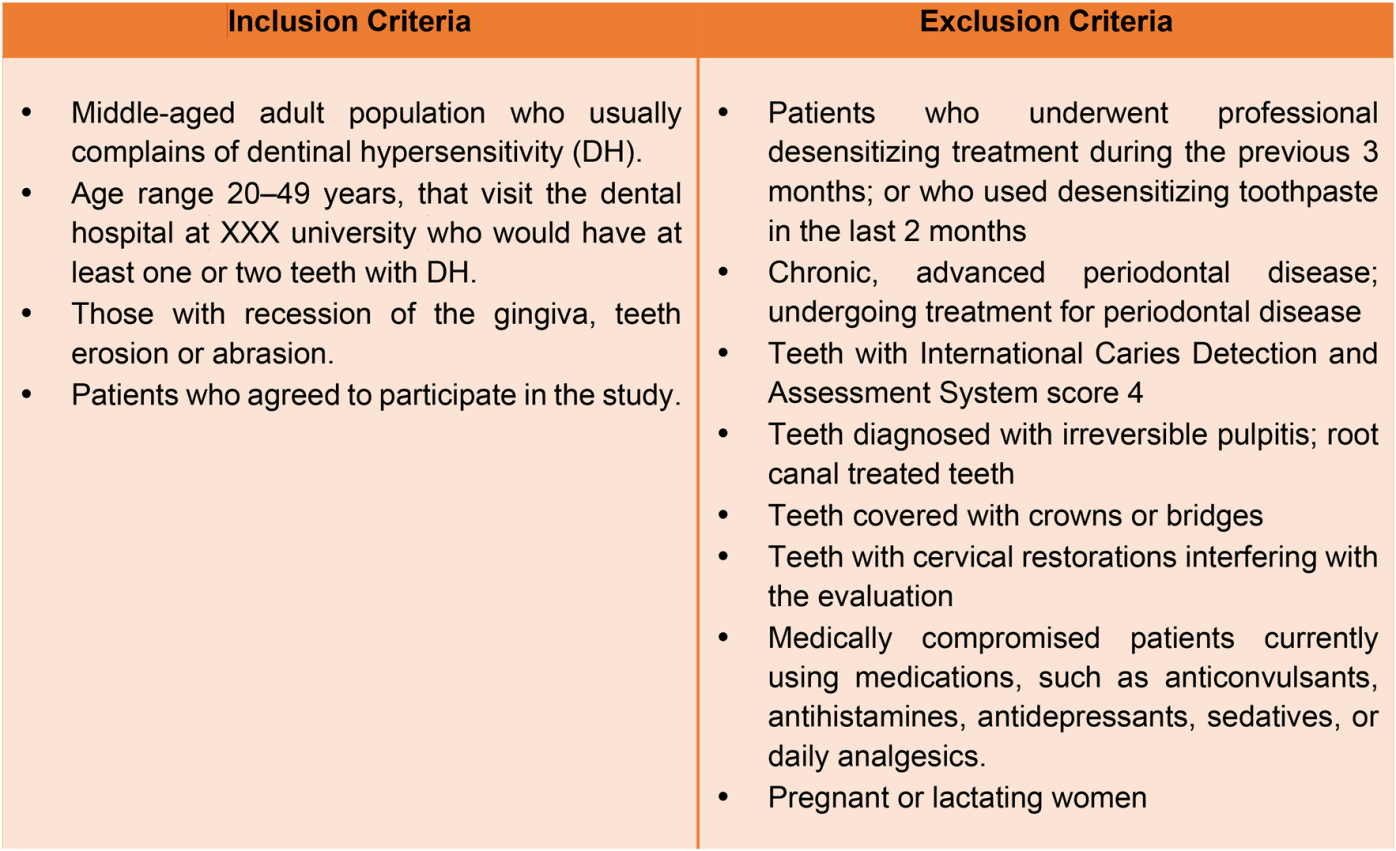
Inclusion and exclusion criteria.

Fifty-five patients (25 males, 30 females) attending the dental hospital, with an age range of 20–49 years, were enrolled in the study. The main examiner clinically evaluated all participants to confirm that they had DH. The inclusion and exclusion criteria are stated in Fig. 2.

### Limitations

An important limitation to the clinical application of the findings is that patient’s follow-up times were limited up to one month instead of longer time follow-up due to COVID-19 pandemic and associated lockdown which lead to discontinuation of the patient’s follow-up visits.

### Randomization

**Figure 3:**
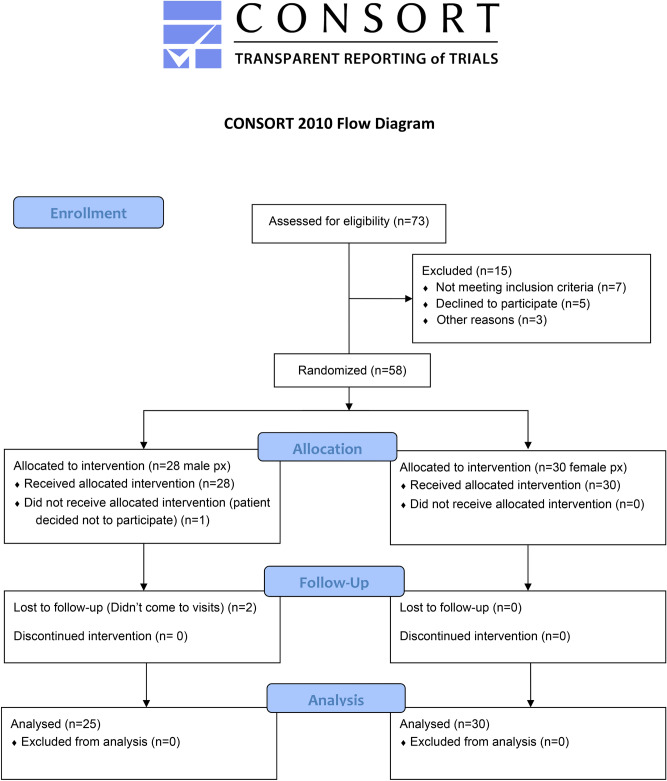
CONSORT Flow Chart.

Seventy teeth from the 55 patients who met inclusion and exclusion criteria and completed all follow-up visits up to one month follow up were incorporated in the study as presented in consort flow chart (Fig. 3) and were randomly assigned to one of three treatment groups: Gluma (n = 24); fluoride varnish (n = 23); and Tetric N-Bond one-step self-etch system (n = 23). For blinded randomization of each tooth to treatment groups, we used the fish-bowl method which was generated by assigning a number presented by a dental intern practicing at the dental hospital.

### Blinding

Each patient was assigned a number after signing the informed consent form. An investigator, who was blinded to the randomization process, recorded DH at baseline (pretreatment), and immediately, at 2 weeks, and at 1 month (30 days) post-treatment. All sensitivity tests were executed by a single experienced examiner who examined the response to tactile, cold, and air blast stimuli. An experienced dentist (who attended a calibration session) accompanied the investigator while recording the responses. All participants were instructed to use a prescribed non-fluoridated toothpaste and to not use any mouth wash during the study period.

### Evaluation of hypersensitivity

Three stimuli were used to assess DH: tactile, cold, and air blast stimuli. The tactile stimulus was examined using an explorer (# 17/23), passing at a right angle to the bucco-cervical tooth surface of concern. Contributors evaluated participants’ pain score on a 10-point visual analogue scale (VAS) which constitutes of a long horizontal line, in which the patient pointed out their pain using a pain scale, similar to the Faces Pain Scale^15^, ranging from no pain (utmost left end) to worst imaginable pain (utmost right end).

For air blast stimuli, air was delivered by a three-way syringe from a typical dental unit air syringe at 40 psi (± 10 psi) and 70 °F (± 5 °F). The air flow was aimed at the tooth surface of concern, for 1 s, from a distance of 1 cm. For cold hypersensitivity assessment, the tooth was isolated using cotton rolls; then, a few drops of extremely cold water were delivered to the tooth from a syringe that had previously been cooled. The cold stimulus scores were assessed by the Schiff Cold Air Sensitivity Scale 18.

### Interventions

All teeth requiring treatment were isolated from adjacent teeth using cotton rolls. In the first group (G1, *n* = 24), Gluma was applied using a mini brush applicator tip onto the exposed hypersensitive tooth surface requiring treatment and was allowed to dry for 60 s. Then the patient was instructed to rinse gently with water. For the second group (G2, *n* = 23), allocated teeth were isolated using cotton rolls and dried with gauze, before applying a thin layer of fluoride varnish to the surface of the tooth using an applicator. The varnish set rapidly, and thereafter moisture contamination was not a concern. Patients were advised to not brush or floss their teeth for a couple of hours and to abstain from hot beverages. In the third group (G3, *n* = 23), Tetric N-Bond self-etch adhesive was applied according to the manufacturer’s instructions, in a thick layer, for at least 30 s, using a light brushing motion. Then, it was dried with a steady stream of air for 3 s and light-cured for 10 s.

DH was assessed for tactile, cold, and air blast stimuli immediately after completion of treatment. Subjects were also recalled for re-evaluation at 2 weeks and at 1 month after desensitizing treatment. Treatment by desensitizing solutions was done by the main examiner; group assignment of participants was hidden from the other examiner, who evaluated dentin hypersensitivity.

### Statistical analysis

Data for the three groups are normally distributed and the variances are equals. As a result, after verifying the assumptions, results were analyzed using analysis of variance (ANOVA), for intergroup comparisons (Gluma, Tetric N-Bond self-etch adhesive, and Flouride varnish). Next, we implemented paired t-test for intragroup comparisons. We applied the scheffe method as post-hoc to determine which treatments are significantly different. Additionally, we used Bonferroni correction to solve the multiple comparisons problem in scheffe method and paired sample t-tests. For all multiple comparison tests we calculated adjusted α equals 0.05/3 = 0.0167 (Bonferroni correction) to compare it with the p-value for all three comparisons. The results are mentioned in the following section.

### Ethical approval

All procedures performed in this study involving human participants were in accordance with the ethical standards of King Abdulaziz university research committee at the faculty of dentistry, Jeddah, Saudi Arabia; approval Id # 129-09-19, and with the 1964 Helsinki declaration and its later amendments or comparable ethical standards.

### Informed consent

For this type of study, informed consent was obtained from all individual participants included in the study.

## Results

All 55 patients completed all appointments up to the 1-month follow-up (Table 1). Participants were recruited from November 1st 2019 up to December 20th 2019. The trial started on January 10th 2020 and the last follow up visit was on February 9th 2020. No side effects or patient concerns/discomfort were reported during the study interval. The study ended on February 9th 2020, being the final day for the last data to be collected from patients on one month follow up visit. Thereafter, data analysis and interpretation were done.

**Table 1:** Patient assignment based on age and sex.

Variable	Number	Percentage
**Sex**		
Male	25	45.5
Female	30	54.5
**Age (years)**		
20–29	16	29.0
30–39	21	38.2
40–49	18	32.7

The intergroup comparison between Gluma, fluoride varnish, and Tetric N-Bond one-step self-etch system for facio-cervical DH after tactile stimulation is displayed in Table 2.

**Table 2:** Comparison of cervical hypersensitivity scores between Gluma®, fluoride varnish, and Tetric® N-Bond one-step self-etch system for tactile stimuli.

Tactile test	Gluma® G1 (Mean ± SD)	Fluoride Varnish G2 (Mean ± SD)	Tetric® N-Bond one-step self-etch system G3 (Mean ± SD)	Significance
Baseline	0.83 ± 0.20	0.84 ± 0.24	0.84 ± 0.22	*P* = 0.98
Immediately	1.24 ± 0.28	1.94 ± 0.31	1.64 ± 0.31	*P* = 0.00*
Two weeks	1.02 ± 0.30	1.90 ± 0.24	1.42 ± 0.32	*P* = 0.00*
One month	0.94 ± 0.24	0.83 ± 0.12	0.80 ± 0.15	*P* = 0.06

G1: Gluma® group; G2: fluoride varnish group; G3: Tetric® N-Bond one-step self-etch system group.

At baseline, all three groups recorded similar scores with no significant difference in DH in response to tactile stimuli (*P* = 0.98). On the other hand, immediately post-treatment and at the 2-week follow-up visit, a significant difference was recorded (*P* = 0.00) among the three groups. After implementing the scheffe method analysis we found that there is a significant difference between Gluma and Tetric N-Bond one-step self-etch system (*P* = 0.00), and Gluma fluoride varnish (*P* = 0.00) immediately and two weeks post treatment. As a result, Gluma (G1) showed the most marked reduction in VAS score of cervical sensitivity, followed by the Tetric N-Bond one-step self-etch system group (G3), while the lowest score was seen for the fluoride varnish group (G2). At the 1-month follow-up visit, all groups showed a similar effect for DH, with no significant difference (*P* = 0.22).

Table 3 presents the intergroup comparison of cervical hypersensitivity scores between Gluma, fluoride varnish, and Tetric N-Bond one-step self-etch system for cold stimulus.

**Table 3:** Comparison of cervical hypersensitivity scores between Gluma®, fluoride varnish, and Tetric® N-Bond one-step self-etch system for cold stimuli.

Cold stimulus Test	Gluma® G1 (Mean ± SD)	Fluoride Varnish G2 (Mean ± SD)	Tetric® N-Bond one-step self-etch system G3 (Mean ± SD)	Significance
Baseline	2.36 ± 0.32	2.11 ± 0.52	2.38 ± 0.42	*P* = 0.06
Immediately	3.63 ± 0.41	5.25 ± 0.46	4.45 ± 0.52	*P* = 0.00*
Two weeks	3.84 ± 0.36	5.01 ± 0.48	5.67 ± 0.18	*P* = 0.00*
One month	2.72 ± 0.28	2.33 ± 0.34	2.57 ± 0.22	*P* = 0.06

G1, Gluma® group; G2, fluoride varnish group; G3, Tetric® N-Bond one-step self-etch system group.

For all three groups at baseline, hypersensitivity scores to cold stimulus were similar, with no significant difference (*P* = 0.06). After implementing the scheffe method analysis we found that there is a significant difference between Gluma and Tetric N-Bond one-step self-etch system (*P* = 0.00), and Gluma and fluoride varnish (*P* = 0.00) immediately and two weeks post treatment. As a result, immediately and 2 weeks after treatment, Gluma treatment resulted in a statistically significantly marked reduction in the Schiff Cold Air Sensitivity Scale 18 for cervical hypersensitivity, as compared to fluoride varnish and the self-etch adhesive (*P* = 0.00). However, the difference among groups was not statistically significant at 1-month post-treatment (*P* = 0.06).

The intergroup comparison between Gluma, fluoride varnish, and Tetric N-Bond one-step self-etch system for facio-cervical DH following evaluation of air-blast stimulus is displayed in Table 4.

**Table 4:** Comparison of cervical hypersensitivity scores between Gluma®, fluoride varnish, and Tetric® N-Bond one-step self-etch system for air-blast stimuli.

Air-blast test	Gluma® G1 (Mean ± SD)	Fluoride Varnish G2 (Mean ± SD)	Tetric® N-Bond one-step self-etch system G3 (Mean ± SD)	Significance
Baseline	0.98 ± 0.71	0.94 ± 0.42	1.23 ± 0.34	*P* = 0.12
Immediately	2.24 ± 0.73	2.92 ± 0.34	2.84 ± 0.28	*P* = 0.00*
Two weeks	1.85 ± 0.45	2.90 ± 0.44	2.45 ± 0.11	*P* = 0.00*
One month	0.84 ± 0.18	0.85 ± 0.12	0.90 ± 0.14	*P* = 0.34

G1, Gluma® group; G2, fluoride varnish group; G3, Tetric® N-Bond one-step self-etch system group.

Similar to tactile and cold stimuli tests, at baseline, all three groups recorded similar scores for DH to air-blast stimuli, with no significant difference (*P* = 0.12). On the other hand, immediately and at the 2-week follow-up visit, there was a significant difference (*P* = 0.00) among the three groups. After implementing the scheffe method analysis we found that there is a significant difference between Gluma and Tetric N-Bond one-step self-etch system (*P* = 0.00), and Gluma and fluoride varnish (*P* = 0.00) respectively immediately and two weeks post treatment. As a result, the Gluma group (G1) showed the most reduction in the Schiff Cold Air Sensitivity Scale 18 score of cervical sensitivity to air-blast stimuli, followed by the self-etch adhesive group (G3), and the fluoride varnish group (G2) consequently. At the 1-month follow-up visit, all groups showed almost similar DH, with no significant difference (*P* = 0.34). Intragroup comparison for tactile, cold, and air-blast stimuli for the Gluma group (G1) showed a statistically significant difference from baseline to every time-point immediately, two weeks and one month post-treatment (*P* = 0.00), whereas the self-etch adhesive showed significant differences in scores from baseline to immediately and to 2-weeks post-treatment (*P* = 0.013), while scores from baseline to 1 month were not significantly different (*P* = 0.06). Similarly, fluoride varnish revealed a statistically significant reduction in cervical DH from baseline to immediately after treatment and from baseline to 2 weeks thereafter (*P* = 0.015). However, there was no significant difference from immediate to 2 weeks and from baseline to 1 month after treatment (*P* = 0.08; Table 5).

**Table 5:** Intragroup comparisons for Gluma®, fluoride varnish, and Tetric® N-Bond one-step self-etch system for tactile, cold, and air-blast stimuli.

	Tactile Stimulus				Cold Stimulus			Air-blast Stimulus		
	Gluma	Fl-V		Tetric	Gluma	Fl-V	Tetric	Gluma	Fl-V	Tetric
**Baseline—immediately**										
Mean	0.39* (0.00)	0.31* (0.015)		1.21* (0.012)	1.39* (0.00)	1.33* (0.016)	2.01* (0.012)	0.38* (0.00)	1.33* (0.014)	1.12 * (0.013)
SD	0.24	0.22		0.23	0.35	0.43	0.26	0.24	0.32	0.32*
**Baseline—2nd week**										
Mean	0.18* (0.00)	0.85* (0.016)		0.21* (0.014)	0.88* (0.00)	2.17* (0.016)	1.22* (0.013)	0.20* (0.00)	1.01* (0.015)	0.42* (0.015)
SD	0.21	0.21		0.14	0.13	0.46	0.44	0.25	0.24	0.18
**Baseline—1st month**										
Mean	0.11* (0.012)	− 0.10		− 0.07	0.79* (0.013)	− 0.15	0.17	0.14* (0.013)	− 0.07	-0.09
SD	0.17	0.13		0.21	0.17	0.33	0.11	0.36	0.22	0.23

*P < 0.05.

## Discussion

Cervical DH is a globally prevalent condition, which is increasing gradually in frequency and severity across different age categories. This may be because the percentage of the population with natural dentition is increasing due to the concurrent rise in life expectancy. The phenomenon of DH is subjective and hence difficult to treat. It is typically evaluated using a pain rating scale, such as the VAS, in different studies^11,16^. Treatments typically have temporary effects. Accessible treatment options currently include the use of topical desensitizing solutions, which can be self- or professionally applied^17^. These treatment modalities utilize tubule-occluding agents^18,19^, sealing of the tubules^20^, and very recently, the use of lasers^21,22^. In this randomized, double-blind, controlled clinical trial, we evaluated the clinical efficacy of Gluma (Heraeus Kulzer), fluoride varnish and a self-etch bond for alleviating DH instantly and over a 1-month period after one direct topical application.

Several studies have shown the effectiveness of desensitizing agents, particularly Gluma, in relieving DH. Gluma effectively reduces dentin hypersensitivity by minimizing dentinal permeability and occluding the peripheral dentinal tubules. This inhibits the fluid flow through the tubules, which is the cause of sensitivity. It also has an antimicrobial effect. Similar to our present study, Yu et al*.* in 2010 conducted a study on the effectiveness of single-bottle self-etching bonding and dentin desensitizing agents, and found that they significantly relieved cervical dentinal sensitivity caused by periodontal surgery, instantly as well as over a period of 30 days following treatment^23^. In another study by Gowri and Kannan, Gluma and Duraphat desensitizers were compared in 38 patients with DH. They used a VAS score at 5 min and at 7-days follow-up and found no significant difference between the scores for Gluma and Duraphat desensitizers, as both significantly reduced DH. However, Gluma showed a significant reduction in VAS scores at 1 week, different from Duraphat. They concluded that Gluma minimized DH more significantly than Duraphat at 1 week after application, and that both desensitizers were effective in reducing DH^24^. Moreover, the efficacy of D/Sense and Gluma in relieving dentinal sensitivity after non-surgical periodontal treatment was evaluated by Al-Qahtani in 2019; forty-five healthy patients after perio-scaling and root planing were enrolled in a double-blind split-mouth study. Teeth were divided and VAS was used to assess hypersensitive scores after tactile and cold stimuli, before periodontal therapy, and then 1, 2, 4, and 6 weeks after periodontal therapy. After a 7-, 14-, and 28-days follow-up period, Gluma reduced the DH significantly, as compared to D/Sense, but after 6 weeks’ follow-up, both Gluma and D/Sense produced the same results. The study concluded that Gluma is a more effective desensitizer during the early follow-up period^25^.

Idon et al. performed a randomized clinical trial in 2017, in which they compared the effects of Gluma, Pro-Relief, and Copal F in relieving DH. The materials were applied to 127 teeth from 68 patients suffering from hypersensitivity. They evaluated tactile and evaporative stimuli utilizing a VAS. After desensitizing solutions were applied, patients were recalled to evaluate DH at 10 min, and 7, 14, and 28 days. Gluma reduced DH more markedly at 10 min after application and also at 1-month post-treatment than the other desensitizing agents. They concluded that Gluma was the in-office agent of choice when treating DH^26^. Unlike our study, another randomized controlled trial, by Verma et al., compared the efficacy of two different desensitizing agents: Gluma and oxalate-containing desensitizer BisBlock. They evaluated the teeth immediately after treatment, and after 1 day, 1 week, 1 month, and 3 months after application. They found a statistically significant reduction in the mean scores after application of Gluma and BisBlock desensitizers at all time-points. BisBlock showed a statistically significantly greater reduction in DH at 7 days and 4 weeks after an evaporative stimulus. They concluded that compared to Gluma, BisBlock yielded a statistically significantly greater reduction in hypersensitivity^27^. As in Verma et al.’s study, another study by Srinivasan-Raj et al. in 2014 found that 8% Arginine paste relieved DH instantly and more effectively than Gluma, and maintained this for a 1-month period^28^. In our study, intergroup comparisons revealed a marked reduction in the hypersensitivity score immediately after, and at a 2-week follow-up for all three groups. Gluma showed a statistically significant and marked minimization of the VAS and Schiff Cold Air Sensitivity Scale 18 score for cervical dentin hypersensitivity when compared to fluoride varnish and self-etch adhesive, immediately after applying the solutions and at the 2-week follow-up visit. At the 1-month follow-up, the Gluma group revealed a non-significantly greater reduction in the VAS score. Another study supporting our finding was published in 2017 by Hajizadeh et al., who tested the efficacy of Gluma desensitizer, Clearfil S3 Bond, and a single-bottle bonding agent in reducing dentin sensitivity; they included 90 teeth from 13 patients. They treated teeth with one of these agents after a periodontal procedure and evaluated the difference in comparison to a water (placebo) control group. They evaluated DH at baseline, 1 day, 7 days, and 4 weeks after application based on VAS, utilizing air stimuli. Similar to our study’s results, all groups resulted in a significant reduction of cervical dentin sensitivity at baseline, but after 7 days and 4 weeks, the single-bottle self-etch bonding agent showed no significant reduction of cervical dentin sensitivity as compared to Gluma, which showed a significant reduction in cervical dentin sensitivity after 1 month of follow-up^29^. In 2012, Brahmbhatt et al. performed a randomized, double-blind, split mouth study to compare 2% NaF solution, Gluma, and NaF-iontophoresis against distilled water (placebo). They evaluated 260 teeth of 25 patients that were randomly assigned to groups. VAS was used to evaluate the pain response by tactile, air, and cold stimuli. Similar to our study, they found that all solutions showed marked reduction in DH at baseline, 15 days, 1 month, and 3 months. However, they found that Gluma and NaF-iontophoresis showed the most reduction when compared to other agents in reducing DH at all time-points. In their study, it was concluded that all three agents reduced sensitivity more than placebo^30^. In could be the limitation of our study that we did not compare agents to a placebo like water but instead we compared three different solutions which are claimed to reduce dentine sensitivity to each other. One of these solutions was Gluma, a true desensitizing agent against a self-etch adhesive and fluoride varnish.

## Conclusion

In conclusion, the current clinical trial proved a marked reduction in DH, with no side-effects reported by patients in any of the three groups. Gluma desensitizing agent showed the best reduction in terms of both VAS and Schiff Cold Air Sensitivity Scale 18 scores over the short period of a 1-month follow-up, after a single direct topical application. Similar scores were found among all three groups at the 1-month follow-up. Gluma can be of great use in the daily dental practice due to its effective relief of DH.
